# Rapid advance of two mountain glaciers in response to mine‐related debris loading

**DOI:** 10.1002/2015JF003504

**Published:** 2015-07-27

**Authors:** Stewart S. R. Jamieson, Marek W. Ewertowski, David J. A. Evans

**Affiliations:** ^1^ Department of GeographyDurham UniversityDurhamUK

**Keywords:** glacier advance, supraglacial debris, human impact, mining, ice deformation, debris‐covered glacier

## Abstract

Rapid glacier advance is known to occur by a range of mechanisms. However, although large‐scale debris loading has been proposed as a process for causing rapid terminus advance, it has rarely been observed. We use satellite remote sensing data to observe accelerated glacier terminus advance in response to massive supraglacial loading on two glaciers in Kyrgyzstan. Over a 15 year period, mining activity has led to the dumping of spoil of up to 180 m thick on large parts of these valley glaciers. We find that the termini of these glaciers advance by 1.2 and 3.2 km, respectively, at a rate of up to 350 m yr^−1^. Our analysis suggests that although enhanced basal sliding could be an important process, massive supraglacial loads have also caused enhanced internal ice deformation that would account for most, or all, of the glacier terminus advance. In addition, narrowing of the glacier valley and mining and dumping of ice alter the mass balance and flow regime of the glaciers. Although the scale of supraglacial loading is massive, this full‐scale experiment provides insight into glacier flow acceleration response where small valley glaciers are impacted by very large volumes of landslide debris.

## Key Points

- Mining spoil is dumped on two glaciers in Kyrgyzstan
- The glaciers advance rapidly due to massive supraglacial debris loading
- Glacier behavior is an analogue for large landslides impacting small glaciers

## 1 Introduction

Rapid glacier speed‐ups and associated terminus advances have been widely attributed to either hydrological [*Kamb et al.*, 1985; *Kamb*, 1987; *Raymond*, 1987; *Eisen et al.*, 2005] or thermal [*Murray et al.*, 2000; *Fowler et al.*, 2001] switching, especially in larger valley glacier systems. However, the potential role of large landslides onto the surface of these and smaller glaciers in the initiation of significant advances has not been extensively or systematically investigated despite a handful of historical case studies on landslide‐ice flow linkages [*Sharp*, 1988; *Murray et al.*, 2003; *Frappé and Clarke*, 2007]. We make the case here, based upon artificial supraglacial debris loading by mine spoil dumping, that accelerations and advances of some small mountain glaciers in response to sudden increased debris loads (e.g., from landslides) need to be acknowledged more widely in investigations of glacier behavior in mountain settings.

### 1.1 Landslide‐Induced Ice Flow Acceleration

In the natural environment, the addition of debris to a glacier surface most often occurs as a result of landslides. The impact of rapid increases in supraglacial debris load on mountain glaciers, specifically the catastrophic deposition of landslide debris, has been reported from several settings in which the immediate glacier response was to advance or surge [e.g., *Tarr*, 1910; *Bull and Marangunic*, 1967, 1968; *Reid*, 1969; *Gardner and Hewitt*, 1990; *Deline*, 2005; *Shugar et al.*, 2012]. Various mechanisms have been proposed to explain such events. First, the earthquake shaking often responsible for the landslide activity may have initiated subglacial till failure through excess porewater pressures [*Shugar et al.*, 2012]. Second, the frictional heat created by the landslide may increase ice melt [*Stark et al.*, 2012] and thereby elevate porewater pressures in subglacial till and/or switch subglacial drainage to a linked cavity system. Third, the increased weight of the landslide debris may, in the case of small or thin glaciers, increase the driving stress significantly [*Shulmeister et al*., 2009]. The exact size of glaciers that could be forced to speed‐up by landslide debris loads has been estimated by *Shugar et al.* [2012] by employing calculations of rock avalanche weight, based upon *Vacco et al.* [2010] bulk density estimates of 2400 kg m^−3^, an equivalent to 2.6 m of ice for every meter of debris. They conclude that the increased debris load associated with a rock avalanche onto the 450 m thick Black Rapids Glacier during the Denali earthquake in 2002 would have created less than a 5% increase in deformational velocity in the glacier. Hence, it is likely that immediate speed‐up responses due to debris loading are significant only in smaller alpine glacier systems where the ratio of supraglacial rock mass originating from a landslide versus glacier ice mass tends to be higher.

In order to verify the role of debris loading and to begin to understand the processes involved in loading as a speed‐up mechanism, more observations of prelandslide and postlandslide glacier responses are required. Measurements of both prelandslide and postlandslide glacier velocities [e.g., *Shugar et al.*, 2012] are rare, and therefore, the scales of, and processes behind, landslide‐induced ice flow acceleration remain largely unquantified. Longer time scale responses to supraglacial landslide coverage have been proposed to be those of glacier mass balance changes, whereby ablation is significantly reduced by the addition of a debris layer and negative mass balance trends become temporarily halted [e.g., *Bull and Marangunic*, 1967, 1968; *Deline*, 2005; *Reznichenko et al.*, 2010, 2011; *Vacco et al.*, 2010; *Shugar et al.*, 2012]. More immediate responses by glaciers to sudden additions of large supraglacial debris loads could be related to mass‐induced increases in driving stress. For example, *Reid* [1969] describes the passing of a kinematic wave through the Sioux Glacier in Alaska and concomitant snout thickening 1 year after a landslide fell onto the ice surface. However, a similar event on the Bualtar Glacier, Pakistan Himalaya, is explained by *Gardner and Hewitt* [1990] as an interruption of the glacial hydrological system and thereby indirectly related to the landslide.

### 1.2 Mining in Glacierized Terrain

Understanding the impact of debris upon glaciers is important not only for gaining insight into past and present glacial response to landslides but also in assessing and mitigating the glaciological, environmental, and infrastructural consequences of mining in glacierized terrain. Increasingly, large‐scale mining operations are being developed in glacierized areas, either as glaciers retreat or through and beneath glaciers whilst they are in situ [*Colgan and Arenson*, 2013]. The loss of ice and rock glaciers as a result of mine excavation is a central environmental concern surrounding these developments. For example, in Chile ~1.4 km^2^ of rock glaciers have been destroyed or degraded at the Sur‐Sur mine since 1980 [*Brenning*, 2008]. Moreover, the excavation of glacier ice has been shown to be necessary for maintaining open mine pits which access resources that were previously subglacial [*Eyles and Rogerson*, 1977; *Colgan*, 2014], causing loss of ice from an area [*Brenning*, 2008]. With further subglacial excavations proposed in Greenland and Canada [*Citterio et al.*, 2009; *Colgan and Arenson*, 2013], ice excavation may become more commonplace. However, the excavation of both rock and the overlying ice means that significant volumes of waste are produced which must be dumped. Glaciers provide a nearby topographically ideal surface upon which to do so. For example, *Citterio et al.* [2009] suggest that the Arcturus Gletscher in Greenland may be impacted by the accumulation of waste rock if proposed mining goes ahead and significant volumes of ice and rock have already been excavated and supraglacially dumped at the Kumtor mine in Kyrgyzstan where a significant impact upon the behavior of a valley glacier was predicted [*Tadzhibaev and Tadzhibaev*, 2005]. Regardless of any environmental impact, by observing supraglacial mine waste dumping over time it may be possible to gain insight into the behavior of glaciers under significant debris loads. Mining activity may therefore provide a measurable analogue for the impacts of landsliding upon glacier flow, while also providing valuable data that can be considered during planning phases for future mining activities in glacierized regions.

### 1.3 Aim

**Figure 1:**
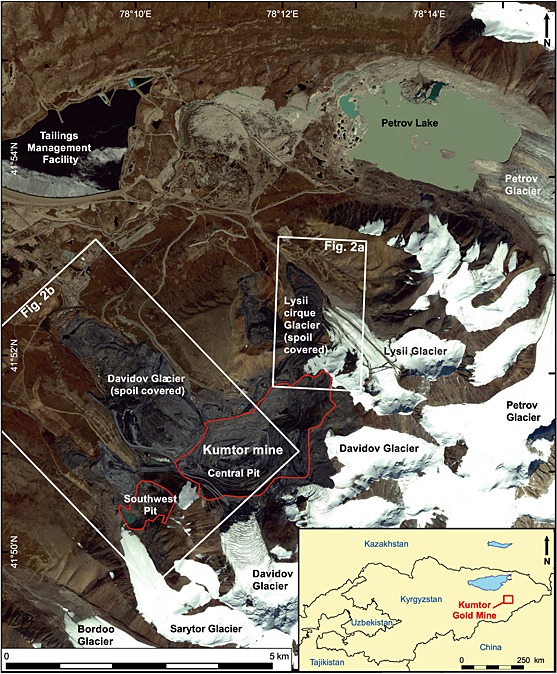
Location map of the Kumtor mine and the glaciers surrounding it in 2014. The background satellite data is a 0.5 m pan‐sharpened image from Digital Globe's WorldView‐2 platform. The inset shows the mine location in Kyrgyzstan.

Given our incomplete understanding of the relationships between supraglacial landslides or mine waste dumping and glacier dynamics, particularly in mountain glacier systems, there is a need to monitor glaciological responses to rapidly increased debris loads. We aim to understand glacier response to rapidly enhanced supraglacial debris loads by observing the example of full‐scale debris loading as a consequence of waste management operations at the Kumtor gold mine in the Akshiirak glacierized massif of the Tien Shan mountains of Kyrgyzstan (Figure 1). *Tadzhibaev and Tadzhibaev* [2005] indicated that the gradual dumping of mine waste on glaciers in this area was likely to have a significant impact upon their flow regime. Mining has continued for 10 years since that study and the dumping of mine waste on the surface of a small unnamed cirque glacier, located above the Lysii valley Glacier (hereon called the Lysii cirque Glacier), and on a larger valley glacier (Davidov Glacier) (Figure 1) provide us with an excellent opportunity to observe and measure such responses.

## 2 Setting

### 2.1 Glacier Changes in the Akshiirak Area

The historical trends in glacier mass balance and marginal positions for the Akshiirak glacierized massif have been assessed by a small number of studies since the late nineteenth century, centered particularly on Petrov Glacier, and have been characterized predominantly by snout recession [e.g., *Sevast'yanov and Funtikov*, 1981; *Kuzmichenok*, 1990; *Dyurgerov et al.*, 1995; *Solomina et al.*, 2004; *Aizen et al.*, 2006; *Jansky et al.*, 2009; *Engel et al.*, 2012]. The area reduction in glacier size for the Akshiirak glacierized massif has been calculated by *Aizen et al.* [2006] to have been 4.2% from 1943 to 1977 and 8.7% from 1977 to 2003, an acceleration that was probably related to increased summer air temperatures since the 1970s. However, a very small number of around 4% of the glaciers in the area advanced during the twentieth century, with some snouts exhibiting surge activity [*Dolgushin and Osipova*, 1982; *Solomina et al.*, 2004; *Aizen et al.*, 2006]. In the area of the Kumtor mine, declassified satellite images show that the Davidov Glacier terminus advanced 240 m in the period 1964–1980. Although *Aizen et al.* [2006] suggest that this occurred after 1977, the satellite images taken in 1973 and aerial photographs taken in 1977 show a steep and crevassed glacier front lying inside an extensive area of ice‐cored moraine produced by earlier downwasting of a debris‐charged snout (see Figure 3b in *Aizen et al.* [2006]), and hence, the advance was already ongoing in 1977. As the region is characterized by continuous mountain permafrost up to 250 m thick [*Redmond et al.*, 2011], the glaciers are likely to be polythermal in character, where thinner marginal ice can freeze to the bed.

### 2.2 The Kumtor Gold Mine

The Kumtor gold mine, which has been quarried since 1997, is located at an altitude of 4000 m above sea level, representing severe environmental conditions for mining. The deep excavations, carried out over an area of ~4.5 km^2^ (as at September 2014) through both glacier ice and bedrock, have necessitated the dumping of rock and ice waste (over 775 million tons by 2012) on surrounding glacier surfaces. Including the dumping of waste on natural surfaces adjacent to the glaciers and areas modified for mine infrastructure, this amounts to ground surface alterations over an area of 9.3 km^2^ [*Redmond et al.*, 2011; *Thalenhorst et al.*, 2012; *Reid et al.*, 2015].

## 3 Data and Methods

### 3.1 Data

In order to examine the changes in glacier dynamics in the vicinity of the mine, we use a range of satellite remote sensing imagery, including declassified images from satellite‐borne camera systems (from 1964, 1973, and 1980), satellite images from Landsat 5, 7, and 8 (covering the 1990–2014 period), satellite images from Aster (from 2002 onward), and very high resolution (VHR) images from IKONOS‐2, QuickBird‐2, GeoEye‐1, WorldView‐1, and WorldView‐2 (from 2002 onward; Table 1). Declassified images enable characterization of the glacial landsystem before mining operations started. Later images demonstrate the extent of mine waste dumping at the same time as the changing extent and character of the glaciers in the area.

**Table 1:** High‐Resolution Remote Sensing Imagery Used to Analyze Glacier Change Between 2002 and 2014^a^

		Resolution (m)		
Acquisition Date	Sensor	Panchromatic	Multispectral	Days Since the Previous Image
30/04/2002	IKONOS‐2	0.82	3.2	0
04/10/2002	QuickBird‐2	0.65	2.4	157
26/02/2003	IKONOS‐2	0.82	3.2	145
01/09/2003	QuickBird‐2	0.65	2.4	187
19/09/2003	QuickBird‐2	0.65	2.4	18
23/03/2006	QuickBird‐2	0.65	2.4	916
14/07/2006	QuickBird‐2	0.65	2.4	113
14/08/2006	QuickBird‐2	0.65	2.4	31
26/09/2009	WorldView‐1	0.46	N/A	1139
03/03/2011	QuickBird‐2	0.65	2.4	523
29/07/2012	GeoEye‐1	0.46	1.8	513
20/10/2012	QuickBird‐2	0.65	2.4	45
29/07/2013	QuickBird‐2	0.65	2.4	290
13/08/2013	WorldView‐2	0.46	1.8	15
05/09/2014	WorldView‐2	0.46	1.8	388

a The column for days since the previous image indicates the temporal spacing of the time series.

### 3.2 Methods

For two glaciers (the Lysii cirque Glacier and the Davidov Glacier; Figure 1), we measure glacier extent from 1998 to 2014 and, after 2002 use the availability of high‐resolution panchromatic and multispectral imagery, to also classify the land/glacier cover for each timeslice. Terminus positions are measured along the centreline of each glacier and are reported relative to the most prominent historical moraines in either valley. The boundaries between material being dumped on, versus adjacent to, the glacier are derived by checking whether spoil terraces overlap the glacier surface. Boundaries between spoil‐related areas and deforming moraine material are determined by the change in color and texture of the material where grey spoil is dumped in terraces and individual dump‐truck loads are visible, and light brown material is being pushed in bands in front of the deforming ice and spoil.

For the Davidov Glacier we also calculate the areal coverage of spoil being dumped either on or next to the glacier, rounded to the nearest 0.01 km^2^. Because the spoil is generally dumped in terraces we are also able to approximate supraglacial debris thickness as it evolves on the Davidov. To do this, we measure the length of a terrace face, and assuming an angle of repose of 32°, we calculate the corresponding vertical thickness of spoil. This assumes that the interface between the base of the spoil and the glacier surface is a horizontal plane. We take five measurements of thickness across the glacier and produce maximum, minimum, and average estimates. We note that this approach is subject to uncertainty which is likely to be on the order of several meters. Sediment volume estimates are calculated by multiplying the thickness by the areal coverage and are rounded to the nearest 0.01 km^3^. This approach allows us to identify links between changes in glacier terminus behavior and the excavation and dumping of mining waste.

For the Davidov Glacier we also calculate how much ice flow would occur via deformation as a block of ice is increasingly loaded over time. The along‐flow variation in the glacier bed, ice surface, and ice thickness is not known at any given time. Therefore, following *Shugar et al.* [2012], deformation flow is computed for a single steady‐state ice column where we make a simplistic assumption that the glacier and its load are of a uniform thickness along flow. Consequently, the results of the calculations should be interpreted with this in mind. For each observation time step, defined by the timing of each VHR satellite image, the ice column thickness is held static and a load equating to either the average thickness of pure rock or of mixed ice and rock is added.

Deformation flow velocity is calculated as v=2An+1 ρgsinαnhn+1. We assume that the density of ice is 917 kg m^−3^, gravitational acceleration (*g*) is 9.8 m s^−1^, and *n* = 3 [*Cuffey and Paterson*, 2010]. Analysis of the Aster digital elevation model (DEM) in this area shows that the average ice surface slope (*α*) is ~5°. The DEM also shows that the elevation difference between the valley floor immediately beyond the glacier snout, and the ice surface 1200 m up glacier at the approximate position of the equilibrium line altitude (ELA) is ~200 m. Assuming a uniform bed slope of 4° (which is the slope of the exposed valley floor in front of the glacier) the bed would rise by 84 m over the same distance. Thus, we calculate that to the nearest 10 m, ice thickness (*h*) is up to 120 m and use this as the uniform value in our velocity calculations. Finally, the temperature of the glacier ice varies through the year, and we therefore generate a range of estimated velocities using ice temperatures (*T*_ice_) of 0°C (*A* = 2.4 × 10^−24^ s^−1^ Pa^−3^), −5°C (*A* = 9.3 × 10^−25^ s^−1^ Pa^−3^), and −10°C (*A* = 3.5 × 10^−25^ s^−1^ Pa^−3^). The density of spoil is greater than that of ice, and following *Vacco et al.* [2010] and *Shugar et al.* [2012], we assume a pure rock bulk density of 2400 kg m^−3^ for our velocity calculations. These are converted to an ice thickness equivalent and added to the underlying glacier thickness (*h*) column. However, the spoil at Kumtor is not always 100% rock but also contains a significant proportion of ice [*Redmond et al.*, 2011; *Thalenhorst et al.*, 2012]. Therefore, we produce a second set of velocity calculations using a lesser bulk spoil density of 1600 kg m^−3^, which is intermediate between that of pure rock and glacier ice.

## 4 Results

**Figure 2:**
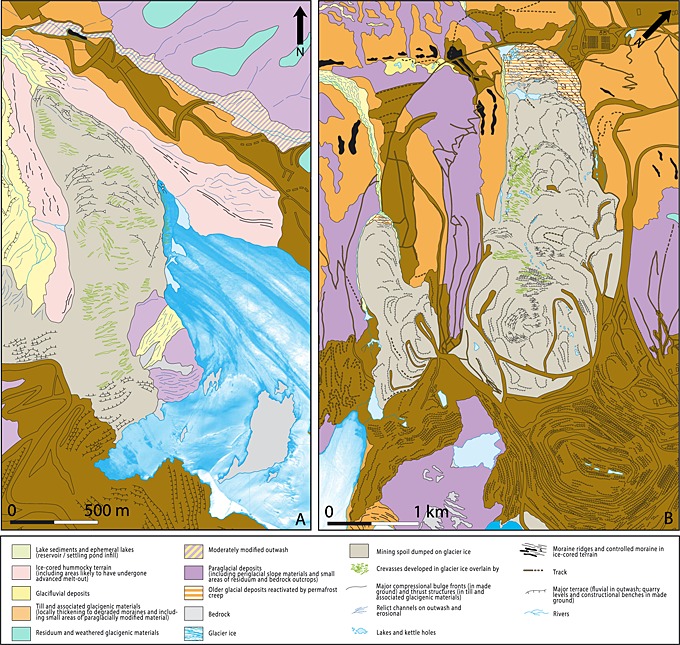
The surficial geology and geomorphology map of the (a) Lysii cirque and (b) Davidov Glaciers and their forelands. The map was compiled from a 0.5 m pan‐sharpened image from Digital Globe's WorldView‐2 platform dating to the 5th of September 2014.

**Figure 3:**
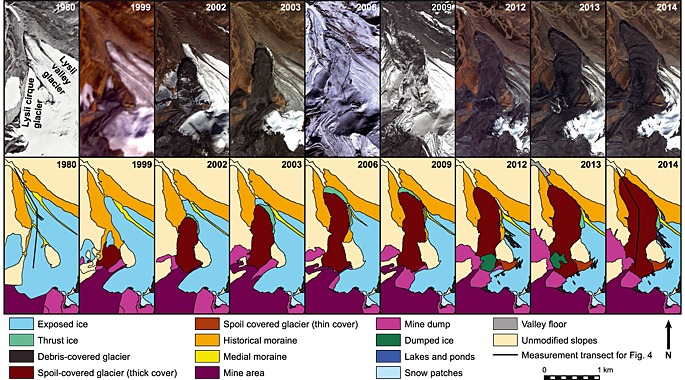
A selected time series of glacier and landcover change for the Lysii cirque Glacier, which hangs above the main Lysii Glacier between 1999 and 2014. Top: high‐resolution satellite imagery from a range of sensors (Table 1). Bottom: classification of surficial geology and glacier ice/debris cover.

Using repeat satellite imagery and their associated landcover classifications (Figures 3, 6, and 7), two particular areas of rapid glacier advance are clearly recorded: a large advance of the Davidov Glacier snout and a significant advance of the Lysii cirque Glacier. Both have been subject to impacts associated with human intervention that include dumping of spoil directly upon or adjacent to the ice and of excavation of glacier ice. Below, we detail the evolution of each of these glaciers, and the associated changes in debris cover characteristics. A detailed surface material map based on the 2014 configuration of both glaciers is shown in Figure 2.

### 4.1 Lysii Cirque Glacier Advance

**Figure 4:**
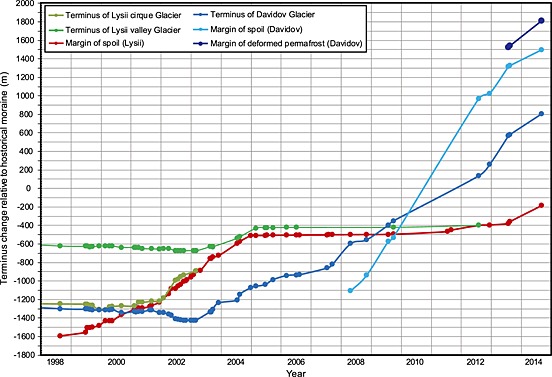
Glacier terminus and spoil margin position for the Lysii cirque Glacier, Lysii valley Glacier, and Davidov Glacier. Dots represent the time of measurement using both high‐resolution satellite imagery and available Landsat and Aster imagery where high‐resolution data were not available. Positions are measured relative to the fronts of the most prominent historical moraines in the Davidov or Lysii Valleys.

Figure 3 records the changes in glacier extent of the small (0.5 km^2^ in 1980) and therefore thin Lysii cirque Glacier and the landcover classification of its immediate surroundings. The glacier hangs above the main Lysii Glacier, and over a 15 year period its terminus advanced ~1.2 km, with much of the movement occurring in the first 6 years (Figure 4). The mine operator refers to the mine waste repository in this area as the Lysii Valley Dump [*Redmond et al.*, 2011], the upper slopes of which form the accumulation basin of the Lysii cirque Glacier. Throughout the observation period, mining spoil consisting of rock and ice was therefore dumped directly onto the head of the glacier.

**Figure 5:**
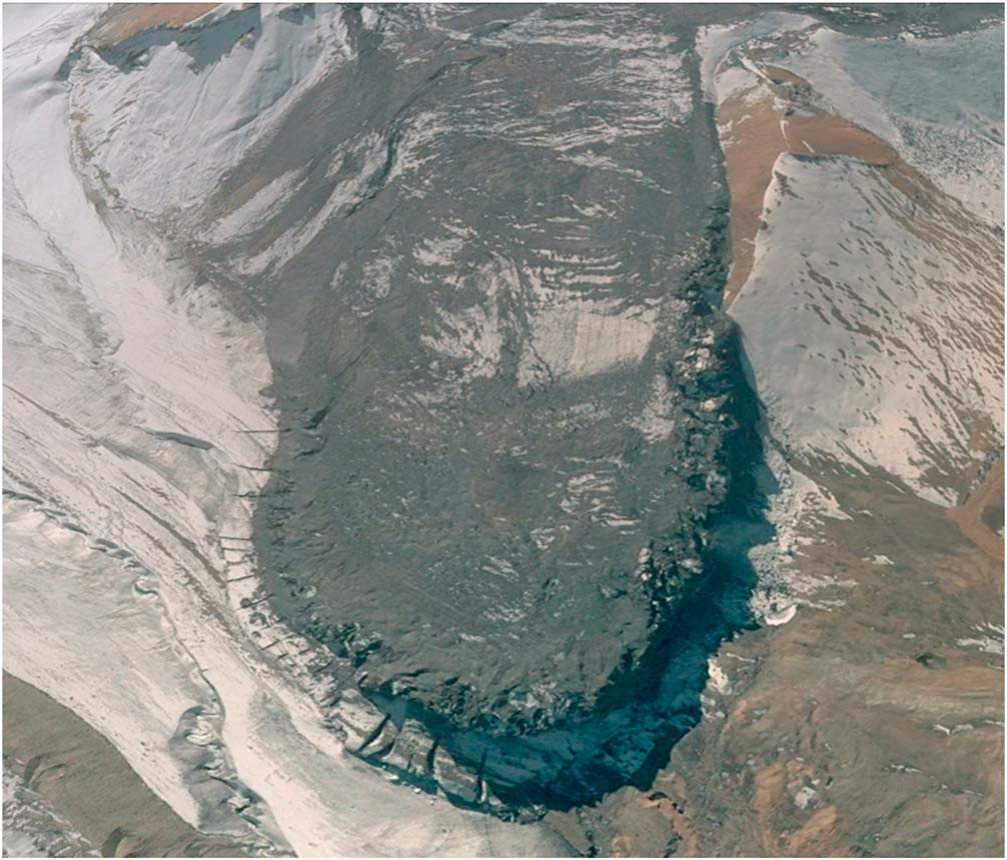
Oblique 2.4 m resolution Quickbird image of the Lysii cirque Glacier front in 2002 during the initial advance of the dual lobate surge front. The stacked debris‐poor and spoil covered lobes and their radial crevasses are clearly visible at this time. For scale, the lobe is 300–350 m wide.

The terminus of Lysii cirque Glacier was in gradual retreat between 1998 and 2002 (Figure 4). In 1999 the majority of the glacier, including the whole of its accumulation area, was covered by newly dumped spoil. However, its terminus was exposed, narrow, and pointed (Figure 3). Between this time period and 2002, the spoil‐covered part of the glacier accelerated downhill and subsequently the glacier terminus comprised the advancing front of this spoil‐covered ice (Figures 3 and 4). Thus, by 2002, a significant advance of the glacier terminus was underway. The glacier terminus comprised a dual lobate structure with an upper, entirely spoil‐covered lobe clearly overriding a lower debris‐poor lobe; both lobes displayed cliffed fronts characterized by densely spaced radial crevasses (Figures 3 and 5). The underlying debris‐poor lobe clearly contained deformed medial moraine debris that could be traced onto the surface of the Lysii Glacier and therefore comprised part of the Lysii Glacier snout that has been buckled, thrust, and steepened by the rapid advance of the Lysii cirque Glacier lobe, which was largely spoil covered all the way up to its accumulation zone. This spoil cover indicated that dumping at the head of the glacier continued throughout the period such that a consistent cover was maintained until extension created some relatively bare ice surfaces in the middle zone of the glacier due to transverse crevassing (Figures 2a, 3, and 5). Figure 4 illustrates the rate at which the glacier terminus advanced during the period of investigation. During 1999–2002 an initial acceleration of the Lysii cirque Glacier front was observed, but no significant change was observed in the Lysii valley Glacier. However, by 2003 the cirque glacier and its associated spoil cover overrode the main trunk of the Lysii valley Glacier causing its terminus to begin advancing. Advance continued through 2003 and 2004, at which point the glacier movement appeared to slow (Figures 3 and 4). By 2004 the glacier terminus had advanced ~700 m, at a relatively constant rate of ~230 m yr^−1^, having previously been in gradual retreat.

As the front of the Lysii cirque Glacier made increasing contact with the Lysii Glacier, the terminus of the Lysii Glacier also advanced by 280 m between 2002 and 2004, surpassing its 1999 extent. Between this time and 2009 the rate of advance of the cirque glacier was slow as it pushed over and onto the Lysii Glacier. Throughout this phase, significant thrusting occurred between the margin of the cirque glacier and the eastern margin of the Lysii Glacier. Over the period 2012‐2014 both glaciers advanced further. However, the advance of the spoil covered component was much faster and it completely overrode the frontal part of the main Lysii Glacier. The entire terminus became spoil covered, as was the trunk of the cirque glacier, indicating once again that dumping had been continued throughout the period of observation. Indeed, the overall area being used to store spoil increased year on year throughout the observation period (Figure 3). In 2012 and 2013 it was also evident that ice, which had been flowing into the top of the main mining pit from the head of the Davidov Glacier and the upper part of the Lysii Glacier [*Redmond et al.*, 2011; *Thalenhorst et al.*, 2012; *Reid et al.*, 2015], had been mined and dumped on the accumulation zone of the cirque glacier. The imagery for 2013 and 2014 shows that within the spoil on the cirque glacier, separate deformation lobes were present and were rapidly transporting spoil mixed with ice toward the toe of the glacier (Figure 3).

Mass input to the glacier could not be quantified for the Lysii cirque Glacier but was orders of magnitude greater than the snowfall accumulation on the glacier, which was likely to be small given its areal extent. Debris delivery to the head of the Lysii cirque Glacier was always sufficient to leave no ice exposed in the area of dumping. Crevasses in the surface of the debris were observed throughout the time period, exposing cleaner ice beneath and attesting to extension in the reservoir zone. The volume of mined ice contained within the spoil steadily increased during and after 2010 as the mine gradually expanded into the accumulation zones of both arms of the Davidov Glacier. Increased rates of ice flow into the pit were documented at this time, and excavators and trucks were used to transport this ice to the various spoil dumps [*Redmond et al.*, 2011; *Thalenhorst et al.*, 2012; *Reid et al.*, 2015].

### 4.2 Davidov Glacier Advance

**Figure 6:**
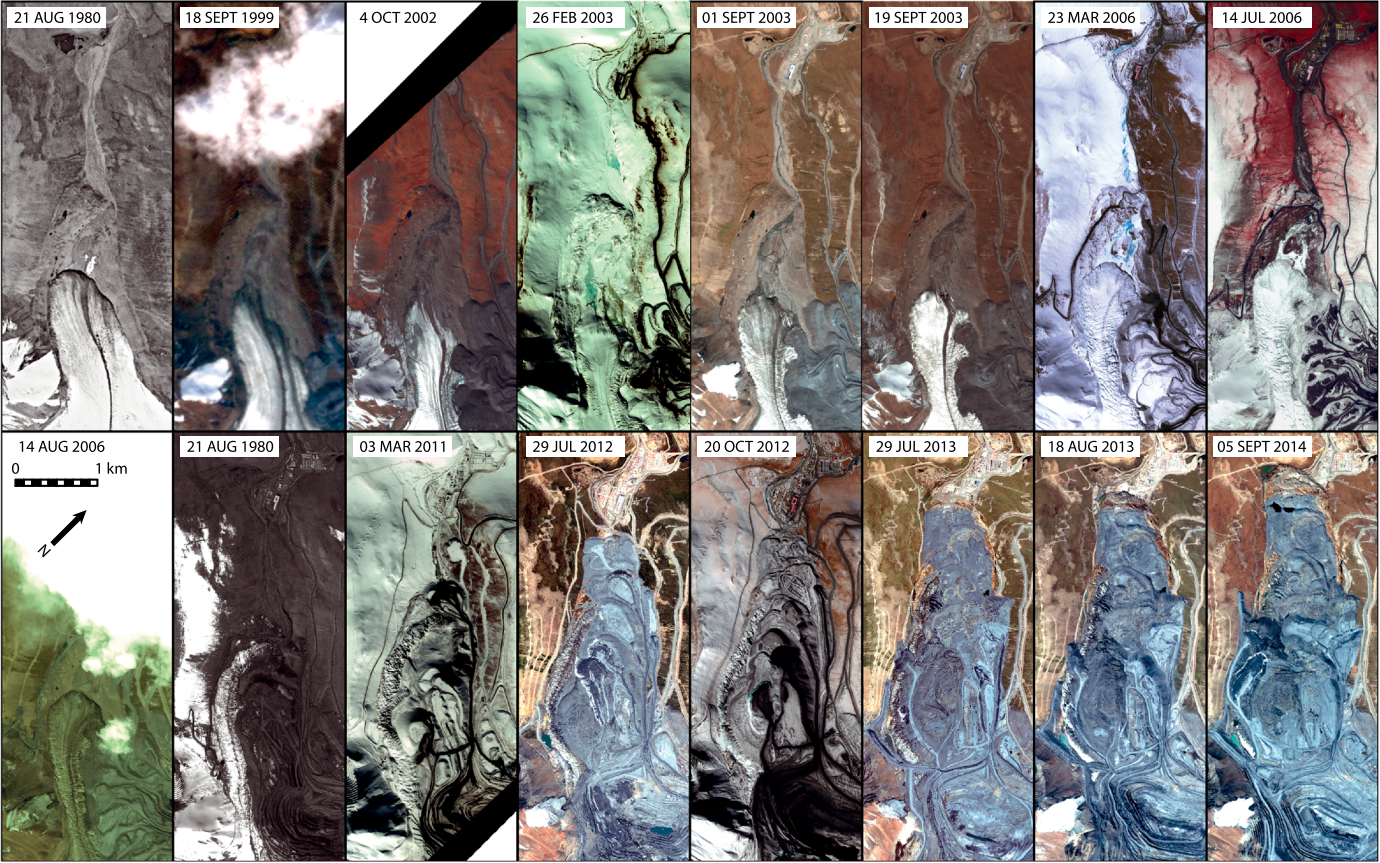
Time series of satellite imagery for Davidov Glacier showing the change in terminus position and the extent of the spoil dumps and mine pit.

**Figure 7:**
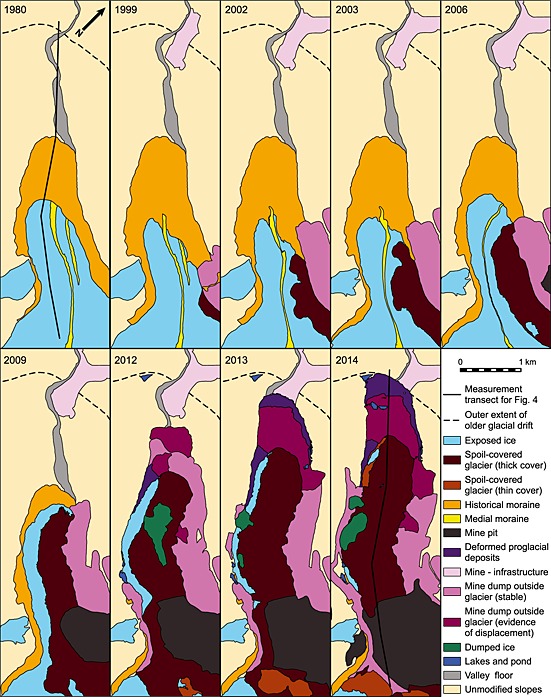
Time series of landcover classification for Davidov Glacier from 1980 to 2014. Additional images are used in the analysis of terminus position (Table 1 and Figures 4 and 6).

**Figure 8:**
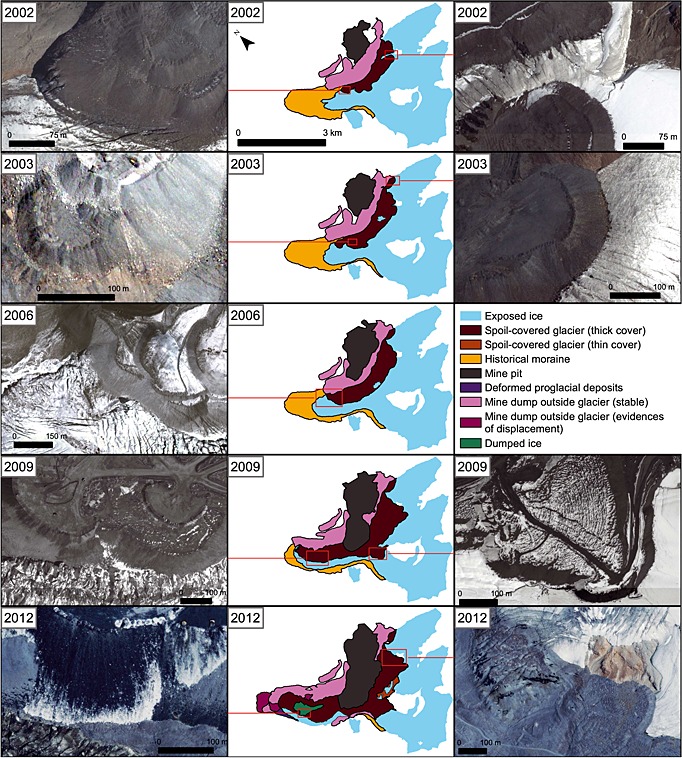
Time series of thin, thick, and laterally dumped spoil distributions in the Davidov Valley. The central panels show the distributions, and the left and right images demonstrate how spoil rests on the glacier in a range of areas. These show that thick spoil is being dumped both at the side and on top of the glacier. The bottom left image from 2012 demonstrates dumping of glacier ice from the trucks.

The Davidov Glacier has three tributary arms in its accumulation area (east, southeast, and south) and at around the elevation of the central mine pit these formerly merged, prior to mining, to form a single trunk glacier. A set of prominent hummocky and pitted moraines was located approximately 1.3 km from the 1999 terminus. Down valley, and up to 2.4 km from the 1999 glacier snout, a further set of more muted moraines of unknown age were evident. Both sets of moraines lay within the limits of the Holocene type IV and V moraines of the region recognized by *Koppes et al.* [2008]. The moraine material in this area has a high ground ice content, and the waste dumps, where not on glacial ice, are on top of permafrost where ice concentrations within the sediment column are up to 40% [*Torgoev and Omorov*, 2014; *Reid et al.*, 2015]. Mine building infrastructure is located directly down valley of the outer extent of this morainic material. During the 15 year observation period, the snout of the Davidov Glacier advanced 2.1 km from its 1999 (Figures 4 and 6 and Movie S1 in the supporting information) position while a policy of dumping spoil next to, and subsequently on to, the glacier was implemented (Figures 7 and 8) [*Redmond et al.*, 2011; *Thalenhorst et al.*, 2012; *Reid et al.*, 2015]. Throughout the period of mining operations, spoil consisted of rock mixed with varying quantities of glacier ice [*Torgoev and Omorov*, 2014]. Table 2 illustrates the evolving spoil thickness and areal extent (and thus its approximate volume) calculated using the imagery. Because the degree of spoil coverage changed the area of the glacier subject to enhanced deformation flow must also have changed accordingly. We calculated and compared the spoil‐loaded ice deformation velocities to that of the unloaded glacier. These velocities, and the percentage increase relative to the unloaded glacier, are shown in Table 3.

**Table 2:** Spoil Thickness, Coverage, and Volume on and Adjacent to the Davidov Glacier From 2002 to 2014 Estimated From High‐Resolution Satellite Imagery (Table 1)

	Supraglacial Spoil					Adjacent Spoil	
	Thickness (m^2^)					Area (km^2^)	
Image Date	Avg	Max	Min	Area (km^2^)	Volume (km^3^)	Valley Side	Proglacial
04 October 2002	44	74	25	0.97	0.04	1.63	0
26 February 2003	36	63	25	1.02	0.04	1.69	0
01 September 2003	52	158	9	1.34	0.07	1.58	0
19 September 2003	57	159	11	1.35	0.08	1.59	0
23 March 2006	63	106	30	1.60	0.10	1.39	0
26 September 2009	55	88	28	2.55	0.14	1.18	0.35
03 March 2011	101	182	18	2.76	0.28	1.18	0.64
29 July 2012	113	157	86	3.25	0.37	1.24	1.05
20 October 2012	105	142	77	3.29	0.35	1.24	1.06
29 July 2013	110	147	48	3.43	0.38	1.29	1.19
05 September 2014	138	186	76	3.27	0.45	1.36	1.31

**Table 3:** Estimated Ice Flow Velocities due to Deformation in Relation to Increased Supraglacial Spoil Dumping^a^

		Rock and Ice Spoil					Rock Spoil				
Image Date	Avg Spoil Thickness (m)	Ice Thickness Equivalent (m)	Velocity (m/yr) *T* = 0°C	% Increase	Velocity (m/yr) *T* = −5°C	Velocity (m/yr) *T* = −10°C	Ice Thickness Equivalent (m)	Velocity (m/yr) *T* = 0°C	% Increase	Velocity (m/yr) *T* = −5°C	Velocity (m/yr) *T* = −10°C
01 December 1999	0	0	3.8	0.0	1.5	0.5	0	3.8	0.0	1.5	0.5
04 October 2002	44	77	27.4	627.0	10.6	4.0	116	56.0	1385.1	21.7	8.2
26 February 2003	36	63	20.4	441.3	7.9	3.0	95	38.5	921.9	14.9	5.6
01 September 2003	52	90	35.7	846.5	13.8	5.2	136	77.8	1962.2	30.1	11.3
19 September 2003	57	99	42.0	1013.9	16.3	6.1	149	95.0	2419.1	36.8	13.9
23 March 2006	63	110	50.9	1250.4	19.7	7.4	165	120.1	3084.1	46.5	17.5
26 September 2009	55	97	40.0	961.4	15.5	5.8	145	89.5	2274.5	34.7	13.1
03 March 2011	101	176	139.0	3585.8	53.9	20.3	264	393.4	10332.7	152.4	57.4
29 July 2012	113	197	184.1	4781.7	71.3	26.8	296	543.4	14313.5	210.6	79.3
20 October 2012	105	183	153.3	3966.1	59.4	22.4	275	440.6	11584.8	170.7	64.2
29 July 2013	110	192	171.3	4443.4	66.4	25.0	287	500.5	13175.1	194.0	73.0
05 September 2014	138	241	310.4	8133.5	120.3	45.3	362	983.1	25975.0	381.0	143.4

a These assume an ice surface slope of 5° and an ice thickness of 120 m. Ice temperatures of 0°C, −5 °C, and −10 °C are applied. Velocities relating to rock and ice spoil versus rock spoil apply bulk spoil densities of 1600 kg m^−3^ versus 2400 kg m^−3^, respectively.

In 1999 the glacier snout appeared narrow and the contributions of ice from each upper arm of the glacier were divided by a prominent medial moraine. The glacier was in recession, particularly on its eastern side. At this time, the spoil from the mine pit started being dumped next to, and on to, the eastern margin of the glacier (Figure 8) in an effort to divert the glacier trunk so that the pit could be expanded further [*Redmond et al.*, 2011; *Thalenhorst et al.*, 2012; *Reid et al.*, 2015]. The dumping of spoil continued into 2002, reaching up to 74 m in thickness (Table 2) with the glacier terminus retreating slightly at the same time as enhanced crevassing became visible in the ice at the foot of the spoil slopes. The position of the medial moraine, which represented the division between flow units coming from each of the glacier tributaries, indicated that the glacier was moving laterally away from the spoil and that the glacier was flowing through a narrower space (Figure 7). Estimated ice velocities at this time were 3.8–0.5 m yr^−1^ depending on *T*_ice_ (Table 3).

At the start of 2003 the shape of the glacier terminus had changed. A bulge of ice could be seen immediately behind the glacier front. The maximum thickness of the spoil had increased to 159 m (Table 2), and the extent of spoil grew, so that by the end of summer it was increasingly encroaching laterally onto the glacier. By the end of 2003, the bulge behind the glacier terminus had gone and the glacier toe had taken on a lobate appearance as it spread to cover more of the valley floor and began to advance. The medial moraine can be observed to have been shifted south and depending on the spoil density and ice temperature, ice velocities estimated for this time were between 6.1 and 95 m yr^−1^ (Table 3).

The next available image of the glacier was for 2006, by which time the toe of the glacier had further spread (Figures 6 and 7), advancing by 350 m since 1999 (Figure 4). The extent of spoil dumping had changed markedly, almost covering half of the width of the glacier trunk compared to its 1999 width. The spoil was also thicker, on average up to 63 m (Table 2) as confirmed by multiple terraces in the dump and the consequence of this is that estimated range for ice flow by deformation increased to between 7.4 m yr^−1^ (*T*_ice_ = −10°C with rock and ice spoil) and 120.1 m yr^−1^ (*T*_ice_ = 0°C with rock spoil; Table 3). By summer 2006 the glacier was much dirtier than in the summer of 2003, perhaps reflecting not only the direct spoil dumping, but the associated dust settling on the ice. Dumping in areas adjacent to the glacier also continues during this period (Figure 8), and the medial moraine has continued to be partially diverted away from the laterally dumped spoil.

After another 3 years, in 2009, the glacier had again advanced significantly to a position only 350 m behind the (Holocene/historical) moraines. Moreover, between 2006 and 2009, some of the existing supraglacial spoil was removed and transported further down‐ice in order to make way for the expansion of the main mine pit, effectively changing the supraglacial debris distribution to cover a much more significant proportion of the glacier (Figures 6 and 7). The glacier itself was only visible from beneath the spoil along its western edge, with approximately 60% of the width of the glacier under the cover of debris. The thickness of spoil was 28–88 m (Table 2). Estimated ice velocities (Table 3) had reduced to 40–89.5 m yr^−1^ (*T*_ice_ = 0°C), 15.5–34.7 m yr^−1^ (*T*_ice_ = −5°C), and 5.8–13.1 m yr^−1^ (*T*_ice_ = −10°C). A major change at this phase of mining operations was the expansion of the pit, at a location just below the ELA, to remove the connection between the eastern arm of the upper Davidov and the main glacier trunk. By this time, ice being formed in the accumulation area was flowing directly into the mine [*Redmond et al.*, 2011; *Thalenhorst et al.*, 2012; *Reid et al.*, 2015] resulting in the need to excavate, transport, and dump increased volumes of ice.

By 2011, the margin of the glacier had advanced yet further and the thickness of spoil on the glacier had again increased (Table 2), further hiding the glacier surface from view. Depending on spoil density, deformation velocities in the ice column (Table 3) are estimated to have been between 139–393.4 m yr^−1^ (*T*_ice_ = 0°C) and 20.3–57.4 m yr^−1^ (*T*_ice_ = −10°C). Spoil dumping was also occurring directly in front of the glacier on top of the historical moraines. The area covered by supraglacial and proglacial dumping grew significantly through 2012 (Table 2), leaving only the narrow western fringe of the glacier margin exposed. Furthermore, the proglacial spoil dump had reached the main deposit of the outermost moraine and was within 180 m of the buildings that were just down valley. By late 2012 the mine pit had expanded and appeared to have completely isolated the ablation area of the original Davidov Glacier from its two accumulating arms. The volume of ice in the spoil increases significantly beyond this time because of the natural flow of the upper arms of the glacier into the mine pit and the subsequent need to mine ice as well as rock to keep the pit operational [*Redmond et al.*, 2011; *Thalenhorst et al.*, 2012]. Maximum spoil thicknesses calculated at this time are ~182 m (Table 2), corresponding closely with profile data from the mine operators [*Thalenhorst et al.*, 2012]. If ice was warm (*T*_ice_ = 0°C), estimated ice velocities were 153.3–440.6 m yr^−1^ depending on spoil, and if ice was cold, velocities were lower at 22.4–64.1 m yr^−1^ (*T*_ice_ = −10°C; Table 3).

In 2013 imagery showed that the spoil dump covered the whole area within the outermost, subdued moraine. At this stage, it was not possible to see the original terminus of the Davidov Glacier. However, the front of the spoil dump had started to flow, spreading into a wide, gradually advancing lobe (Figures 4, 6, and 7). Related to this, it was also apparent that the light brown colored outermost subdued moraine, which was being pushed and compressed into a narrow band in front of the dark grey advancing spoil (Figures 6 and 7). By summer the wave of deforming spoil and moraine had begun bulldozing through the buildings and infrastructure (Figures 6 and 7). Throughout 2013 and 2014, the extent of the mining spoil appears to alter very little. However, the lobes of deforming moraine material advanced another 650 m further down the valley, destroying more buildings (Figures 6 and 7). By 2014, the entire ablation zone of the original Davidov Glacier was buried beneath spoil that we estimate reached up to 186 m thick (Table 2). Estimated ice velocities ranged between 45.3 and 983.1 m yr^−1^ depending on the uncertainties around spoil density and ice temperature (Table 3).

## 5 Discussion

Many glaciers in the Kumtor region are known to be naturally surging systems where it is not unusual to observe rapid glacier advance. However, given the scale and timing of mining activity, it seems likely that the glacier advances described above were triggered by a combination of processes, some of which were not internal to the natural glacier dynamics. The huge scale reorganization of the Davidov Glacier has been responsible for the deterioration of the buildings at the mine due to glacier bulldozing, whereby mining spoil and ice transferred to the ablation zone and ice‐cored moraine has reinvigorated flow in the ablating glacier snout and reactivated the ice‐cored moraine in the lower Davidov Valley. This is effectively a human‐induced glaciological process‐form regime that includes glacier advance, apron entrainment (in the sense of *Shaw* [1977] and *Evans* [1989]), permafrost creep, and proglacial thrust moraine construction. The latter is often used as a diagnostic element of a surging glacier landsystem [*Evans and Rea*, 1999, 2003], but in this case the landforms are related to a single rapid advance. We now discuss the causes of this advance and the implications for understanding the impact of rapid supraglacial loading in natural systems.

### 5.1 Advance by Rapid and Substantial Supraglacial Debris Loading

The observations reported above indicate that an anomalous glaciological process‐response regime, involving rapid glacier advance, was likely put into operation by the emplacement since 1997 of huge supraglacial debris loads onto the surfaces of the Davidov and Lysii cirque Glaciers. The operator has acknowledged that the increased load of the mining waste on the part of Davidov Glacier immediately above the main mine pit has caused accelerated sliding on the till surface below the glacier [*Redmond et al.*, 2011]. In an attempt to remedy this, supraglacial load was removed and redeposited lower down on the glacier, and pumping wells were installed in the till in order to reduce porewater pressures. This was undertaken specifically to “depressurize” the till as well as the bedrock in the quarry face [*Torgoev and Torgoev*, 2014; *Torgoev and Omorov*, 2014]. This demonstrates the influence of the debris load on ice in a location where supraglacial loading was likely far less significant than on the ablation zone of the Davidov Glacier.

A number of mechanisms are likely to be important in altering the behavior of the glacier as the rapid, substantial, and continuous supraglacial mass loading occurred. These include increased glacial mass balance and, consequently, enhanced ice deformation, as demonstrated by our measurements and ice flow calculations. Ice velocities measured on the Davidov Glacier between November 1999 and December 2000 [*Tadzhibaev and Tadzhibaev*, 2005], prior to significant spoil dumping, indicate maximum ice flow velocities of ~17 mm d^−1^ (6.2 m yr^−1^) for a short period during the summer, with average velocities over this period of ~6 mm d^−1^ (2.2 m yr^−1^). This is consistent with our estimate of velocity by ice deformation for this period which is between 0.5 and 3.8 m yr^−1^ (Table 3) depending upon ice temperature. *Tadzhibaev and Tadzhibaev* [2005] also predicted that the enhancement of supraglacial loading will increase strain rates sharply on the glacier, causing ice flow to be enhanced. This is indeed what our observations show, and our calculations suggest that ice flow velocity due to deformation increased significantly during the period of observation (Table 3). As spoil reaches its greatest thickness, ice flow by deformation could account for between 45 and 983 m yr^−1^ of the advance depending on spoil density. Given that the maximum terminus advance rate measured for the Davidov Glacier is ~350 m yr^−1^, the increase in estimated ice velocity via deformation (Table 3) could therefore be invoked to explain all, or at least a significant proportion of the change in flow regime observed at the Davidov Glacier.

Given that yearly average ice temperatures are likely to be below 0°C, we suggest that our estimates for ice deformation under cooler conditions are likely the most relevant at Kumtor. Even at *T*_ice_ = −5°C, there is a gap between observed ice flow and our estimates of ice deformation which suggests that basal processes such as sliding and till deformation are likely to be operating. This is supported by the presence of high water pressures at the ice‐bed interface [*Redmond et al.*, 2011] and by observations that some parts of the waste‐covered glacier may be moving as individual blocks [*Torgoev and Omorov*, 2014]. Basal sliding rates may be modified via two processes. First, if R‐channels exist beneath the Davidov Glacier, one potential impact of the enhanced ice deformation rates would be to accelerate creep closure rates at the base of the ice, altering the effective pressure regime, and therefore sliding speed of the glacier [*Iken and Bindschadler*, 1986]. Second, given the enhanced supraglacial load is the equivalent of adding up to ~360 m of ice to the existing 120 m ice column (Table 3), it may be possible to trigger a switch from a channelized to a linked‐cavity subglacial drainage system assuming that a linked‐cavity system was not already in operation, thus enabling higher basal sliding velocities [*Fowler*, 1987]. Third, the increased pressure at the base of the ice may also increase basal melt rates [*Cuffey and Paterson*, 2010], increasing the likelihood of enhancing effective pressure and thus sliding. The degree of enhancement in basal sliding rates are not calculated here because the effective pressure, a key parameter in the empirical sliding relation for ice flow [*Cuffey and Paterson*, 2010], is unknown due to the lack of available measurements of basal water pressure variation through time in the Davidov Glacier.

Finally, deformation is not only likely to be enhanced in the ice column but also highly likely to be enhanced in the subglacial till because sediment deformation is proportional to basal shear stress and to basal water pressures [*Cuffey and Paterson*, 2010]. As indicated above, basal hydraulic conditions and basal shear stresses are likely to have been altered as a result of the substantial mass loading [*Harrison and Post*, 2003; *Truffer and Harrison*, 2006]. Therefore, given that the base of the Davidov Valley is underlain by sediments containing a high ground ice content [*Torgoev and Omorov*, 2014; *Reid et al.*, 2015], both ice and sediment deformation processes beneath the glacier ice will have contributed to the glacier advance. Early observations of the Davidov Glacier, for example, showed that beyond the toe of the glacier in 1999–2006 (Figure 6) a significant moraine sequence was present which was subsequently overridden by the advancing ice in around 2009. This overriding is coincident with a speed‐up in the advance of the terminus, and we infer that the onset of deformation in this thicker column of till (and in any glacier or permafrost ice within it) may be at least partly responsible.

### 5.2 Glacier Terminus Change by Altered Ice Flow Pathway

Not only has the mass balance of the glacier been significantly altered, but the pathway through which ice travels in the Davidov Valley has changed over time in two significant ways. First, the 2011 mining report [*Redmond et al.*, 2011] indicates that one objective of lateral spoil dumping at the Davidov Glacier was to push the glacier away from the central mine pit. As already indicated, the lateral translation of the glacier is visible in the position of the medial moraine between 1999 and 2006 as the glacier is moved by the addition of spoil at its margin. The opposite margin has not moved, and therefore, the impact of the initial spoil dumping is to cause an effective narrowing in the valley width. Observations and modeling suggest that this causes squeezing of the ice which can take 1–2 years to respond to a particular load change [*Kuzmichenok*, 2012]. Given that the mass balance of the glacier barely changes in this initial phase of dumping, the natural response of the glacier to flowing through a narrowed channel is to accelerate its flow. Because ice discharge is a function of glacier thickness and width, the glacier must have responded to the narrowing of channel width by locally thickening. This would have caused a steepening of ice surface gradient downstream and therefore would have contributed to the acceleration of ice flow velocity and to the advance in terminus position. In the case of the Davidov Glacier, the degree of channel width restriction is unclear because spoil is also dumped on the glacier surface at this time. We suggest that this form of change in effective valley configuration is one that could occur naturally if landslide debris were to be deposited at the lateral margin of a glacier, particularly where a significant cleft is present between the rockwall and the glacier side.

Second, in Kumtor, as predicted for mines in other actively glacierized catchments [*Eyles and Rogerson*, 1977; *Citterio et al.*, 2009; *Colgan and Arenson*, 2013; *Colgan*, 2014], ice has had a significant impact upon the mining operations. As our results and the mining reports [*Redmond et al.*, 2011; *Thalenhorst et al.*, 2012; *Reid et al.*, 2015] show, this necessitated the mining of ice from the eastern lip of the mine pit, and the subsequent dumping of that ice within the main spoil dumps further down the glacier. Consequently, the natural mass transport pathway of the glacier is being circumvented. Under spoil‐free glacier conditions prior to mine development, it might be expected that ice from the area above the mine would take many years to travel past the area that subsequently became the central pit (Figure 1). However, the mining of the ice means that the ice now travels (by dumper truck) up to several kilometers in a matter of minutes to hours. Since this transport is occurring entirely in the ablation zone, the ice is reaching a lower point on the glacier without having undergone the several years' worth of ablation. The consequence of this is that the terminus of the glacier should be expected to advance because the mass balance regime of the glacier has artificially been made less negative in the ablation zone. This process cannot be easily disentangled from the overall influence of the supraglacial spoil which will also act as a thermal insulator. In natural glacier systems this rapid bypassing of most of the ablation zone occurs rarely, for example, where “reconstituted” or “fall” glaciers accumulate at the base of steep bedrock cliffs after ice avalanches from glacier bodies on higher plateaux directly to the floors of adjacent valleys [*Gellatly et al.*, 1986; *Benn and Lehmkuhl*, 2000].

### 5.3 Are the Kumtor Glaciers an Analogue for the Impact of Supraglacial Landslides?

It has been proposed that the rapid addition of supraglacial debris loads by natural landsliding can cause glacier advances and, in surge‐type glaciers, may trigger surges [e.g., *Tarr*, 1910; *Bull and Marangunic*, 1967, 1968; *Reid*, 1969; *Gardner and Hewitt*, 1990; *Deline*, 2005; *Shugar et al.*, 2012]. However, the delivery of volumes large enough and frequently enough to initiate immediate changes in glacier deformation and/or sliding rates that are repeated and are high enough to be classified as a surge is debatable [*Vacco et al.*, 2010; *Shugar et al.*, 2012]. Potential driving mechanisms such as earthquake‐driven subglacial till failure [*Shugar et al.*, 2012] and increased ice melt and elevated till porewater pressures or switches in subglacial drainage networks due to landslide induced frictional heat [*Stark et al.*, 2012] are difficult to test. However, a further hypothesized driver in small glaciers, the weight of the landslide debris increasing the driving stress [*Shulmeister et al*., 2009], has been tested at the Kumtor mine on both the Lysii cirque Glacier and the Davidov valley Glacier by the dumping of large volumes of mine waste. The waste dumping constitutes substantially more volume than normally delivered to glacier surfaces by rock slope failures, leading some to suggest that the Davidov is now operating as a landslide rather than as a glacier [*Torgoev and Omorov*, 2014]. However, it is nonetheless an appropriate upper end‐member analogue for potential landslide‐induced advances of alpine glacier systems where the ratio of supraglacial rock mass originating from a landslide versus glacier ice mass tends to be higher. The Kumtor example can therefore augment the small number of cases, where estimates have been secured for both prelandslide and postlandslide glacier velocities [e.g., *Shugar et al.*, 2012], and constitutes a clear analogue for mass‐induced increases in driving stress with similar glaciological responses to those reported by *Reid* [1969] for the landslide‐induced kinematic wave in Sioux Glacier, Alaska.

## 6 Conclusions

We report observations of evolving glacier terminus position change from the Kumtor mine in Kyrgyzstan between 1998 and 2014. In the area, glaciers adjacent to the mine have been used as spoil dumps. We find not only that glacier ice can have a significant impact upon mining activities, but more importantly, that mining operations can drive significant changes in glacier behavior. We used high‐resolution satellite imagery to map the terminus advance of two glaciers and to map the evolving distribution of mining spoil on the surface of these glaciers. Our findings include the following:

1. The identification of two significant and rapid human‐induced glacier terminus advances.
2. The terminus of the Lysii cirque Glacier advanced by 0.2 km, bulldozing and then overriding the terminus of the larger Lysii valley Glacier. A total advance of 1.2 km had occurred by 2014.
3. The Davidov valley Glacier was subject to dumping of up to 0.437 km^3^ of spoil, resulting in a terminus advance of 3.2 km at a rate of up to 350 m yr^−1^.
4. The main cause of glacier terminus advance is massive supraglacial loading by up to ~190 m of rock and ice spoil. Although basal sliding and till deformation were likely to have become enhanced, calculations of glacier flow suggest that it is possible that a significant proportion of the advance could have been achieved via increased ice deformation rates due to the enhanced load.
5. Terminus advance may also have been linked to artificial narrowing of the glacier flow channel and to altered mass balance conditions due to the mining of upstream ice and the circumvention of the natural flow pathway.
6. The glacier responses to artificial debris loading provide verification that instantaneous additions of substantial supraglacial debris loads (i.e., via rock slope failure and landsliding) can trigger anomalous fast glacier flow and rapid advance.

## Supporting information

Text S1

Movie S1

Movie S1
