# Supplementary material for: Rapid advance of two mountain glaciers in response to mine‐related debris loading
Source: J Geophys Res Earth Surf. 2015 Jul 27;120(7):1418–35. doi: 10.1002/2015JF003504 (PMC4957274; doi:10.1002/2015JF003504)
Supplement: Supplementary file 1 — Text S1 [file JGRF-120-1418-s001.docx]

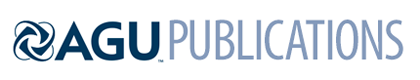


*[Earth Surface]*

Supporting Information for

**Rapid advance of two mountain glaciers in response to mine-related debris loading** Stewart S.R. Jamieson^1^*, Marek W. Ewertowski^1^ and David J.A. Evans^1^

^1^ Department of Geography, Durham University, South Road, Durham DH1 3LE, UK

*Corresponding author: Stewart.Jamieson@durham.ac.uk

**Contents of this file**

Information relating to Movie S1

Captions for Movie S1

**Introduction**

Two movie files (2015JF003504-ms01.avi and 2015JF003504-ms01.gif) act as supporting information to the main article. This supporting information document contains details of the files and details the caption for the movie.

The movies have identical content (and thus are both Movie S1), but are provided in two formats (as an avi and as an animated gif) in order to ensure compatibility with a wide range of computer systems.

The movie iterates through the timeseries of high-resolution satellite imagery used in the analysis of the Davidov Glacier and mine waste dump. The aim of the animation is to provide an accessible way to visualize the changes at the glacier. Details of the data and the approach to analysis are contained in the main manuscript text.

Movie S1. Animated time-series of the changes in behavior and waste dumping at the Davidov Glacier, Kyrgyzstan. Each timeslice relates to a different very high resolution satellite image, with the image date shown in the top left corner.
